# Graft kidney hydronephrosis caused by transplant ureter inguinal hernia: A case report

**DOI:** 10.1097/MD.0000000000025965

**Published:** 2021-05-28

**Authors:** Tian-You Chang, Chao-Hsiang Chang, Ping-Chin Lai, Wei-Ching Lin

**Affiliations:** a Department of Urology, China Medical University Hospital; b The Kidney Institute and Division of Nephrology, China Medical University Hospital; c Department of Radiology, China Medical University Hospital; d School of Medicine, College of Chinese Medicine, China Medical University; e Department of Biomedical Imaging and Radiological Science, China Medical University, Taichung, Taiwan.

**Keywords:** graft kidney hydronephrosis, inguinal hernia, renal transplantation, ureteral inguinal hernia

## Abstract

**Rationale:**

Ureteral obstruction of the graft kidney is a common complication of kidney transplantation. However, ureteral obstruction caused by inguinal hernia has rarely been reported. We present a rare case of ureteral obstruction with allograft dysfunction caused by an inguinal hernia.

**Patient concerns:**

A 76-year-old man, who was a renal transplant recipient, presented with bilateral pitting oedema, reduced urine output, and right inguinal hernia.

**Diagnoses:**

Abdominal computed tomography revealed severe hydroureteronephrosis of the kidney allograft. A right inguinal hernia with ureteral incarceration was observed.

**Interventions:**

The patient underwent graft percutaneous nephrostomy, followed by antegrade insertion of a double-J tube (DJ). Gradual improvement was observed in his renal function. Right inguinal herniorrhaphy was performed 5 days later.

**Outcomes:**

The renal function returned to normal after percutaneous nephrostomy and insertion of the DJ. A right inguinal direct-type hernia with ureter adhesion to the hernial sac was observed during the surgery. The posterior wall defect was repaired by the McVay technique. The DJ was removed after 1 month. The patient's renal function remained stable at 6-month follow-up.

**Lessons:**

The orientation of the graft kidney has a significant influence on the location of the ureter. Upward orientation of the hilum will result in superficial location of the ureter, rendering it close to the hernial sac and susceptible to incarceration. The transplant surgeon should be aware of such a presentation of graft dysfunction with inguinal hernia to prevent a delay in the diagnosis and graft loss.

## 1 Introduction

Ureteral obstruction is a common complication of renal transplantation. However, ureteral obstruction caused by inguinal hernia is a rare complication, and there are limited reports in the literature. The common causes of inguinal herniation of the transplant ureter are redundancy of transplant ureter^[1]^ and anterior positioning of the ureter in relation to the spermatic cord.^[2,3]^ However, these conditions were not observed in our patient. Herein, we present a special case of allograft dysfunction, wherein the transplant ureter inguinal hernia was related to the special position of the graft kidney.

## 2 Case report

A 76-year-old man visited our clinic with complaints of swelling and indentation upon pressing in both legs for 2 weeks. He had past medical history of one-vessel coronary artery disease, biliary stone had undergone endoscopic retrograde cholangiopancreatography, and end-stage renal disease secondary to analgesic nephropathy. He had undergone living-donor kidney transplantation 3 years ago in mainland China.

**Figure 1:**
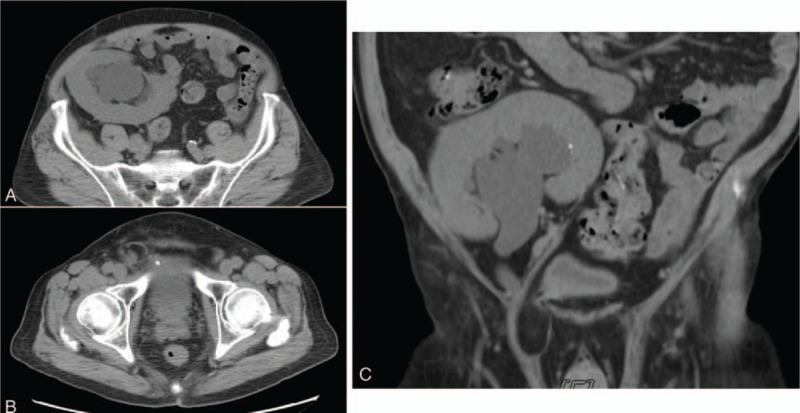
CT scan. (A) The renal hilum of the transplanted kidney is seen facing upwards. (B) The ureter extends superficially downwards towards/into the bladder; however, is compressed by the hernial sac and is therefore entrapped in the inguinal canal. (C) Ureter is compressed by the inguinal hernia.

**Figure 2:**
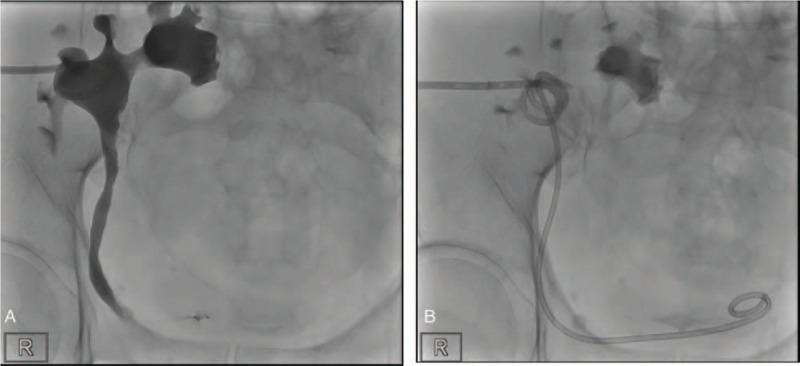
Antegrade pyelography. (A) AP revealing near total obstruction of the ureter at the level of superior margin of the pubic symphysis. (B) DJ is inserted in the antegrade direction. AP = antegrade pyelography.

Physical examination revealed a right-sided inguinal hernia and bilateral lower limbs grade 2/4 pitting oedema. His serum creatinine level increased to 1.2 mg/dL (baseline, 0.8 mg/dL), and bedside renal echography revealed graft kidney hydronephrosis. Abdominal computed tomography was performed, which revealed severe hydroureteronephrosis of the kidney allograft (Fig. 1). External compression of the transplant ureter by the right inguinal hernial sac was observed. In addition, the grafted kidney had an abnormal position, and the hilum was facing upwards. The graft artery and vein measured approximately 6.5 cm and 5.4 cm in length, respectively. Percutaneous nephrostomy and antegrade pyelography were performed considering the deterioration in renal function (Fig. 2). Minimal amount of the contrast medium could reach the bladder. The obstruction was at the level immediately above the pubic symphysis. A double-J tube (DJ) was inserted in the antegrade direction, following which we proceeded with the surgical exploration.

**Figure 3:**
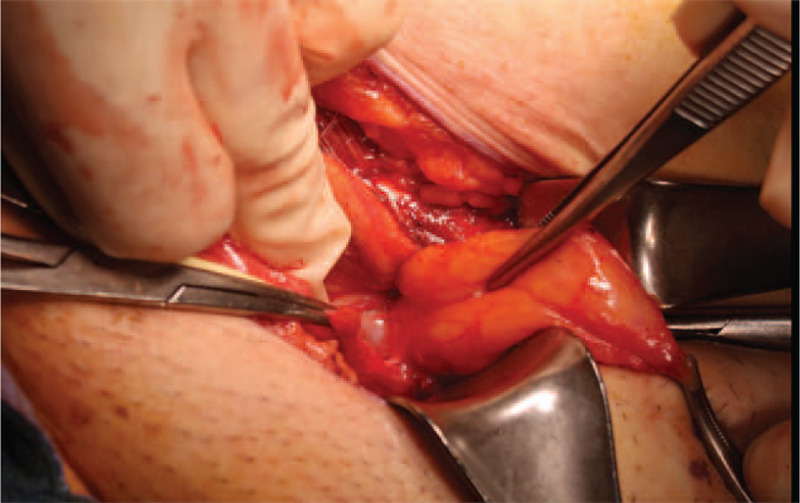
Direct-type hernia seen on the right side of the picture and ureter with DJ is seen external to the hernial sac, located deep and close to its neck.

Through a 7 cm inguinal incision, the spermatic cord and direct-type hernial sac were identified. Owing to preoperative insertion of the DJ, the ureter was identified when we trace the hernial sac down to its neck and the DJ was palpable inside it. (Fig. 3) Adhesion was observed between the transplant ureter and hernial sac. After adhesiolysis, high ligation of the hernial sac was done by the purse-string suture technique. The posterior wall defect was repaired by the McVay procedure with interrupted sutures between the conjoint tendon and Cooper's ligament. Foley catheter and DJ were retained for 2 days and 1 month, respectively. After removal of the DJ, follow-up renal sonography revealed residual hydronephrosis. However, serum creatinine level decreased from 1.2 mg/dL to 0.91 mg/dL, and the bilateral lower limb pitting oedema also disappeared. At 6 months of follow-up, there were no signs of recurrent inguinal hernia and the renal function remained stable.

Written informed consent was obtained from the patient for publication of this report. Since this study used only de-identified patient data and published data from the literature, approval from our institutional review board was not required.

## 3 Discussion

There are many causes of graft kidney hydronephrosis, including ureteral stones, reflux, infection, and rejection. Inguinal herniation of the transplant ureter is a rare cause of ureteral obstruction in cases of kidney transplantation.^[2,4–6]^ Despite the paucity of studies in the literature, this complication should be considered in the differential diagnosis because it can be managed surgically by urologists and transplant surgeons.

Possible risk factors include redundant ureter,^[1]^ anterior positioning of the ureter in relation to the spermatic cord,^[2,3]^ and obesity.^[7]^ However, these factors were not present in our case. In our opinion, the complication was related to the abnormal position of the graft kidney. In our case, the graft kidney had a very long artery (6.5 cm) and vein (5.4 cm), due to which the hilum faced upwards. Therefore, the ureter was located superficially downwards and was prone to inguinal herniation.

Ultrasound is the first-line diagnostic imaging modality. Ultrasound usually reveals hydronephrosis; however, may not enable visualization of the full length of the ureter. computed tomography can provide information on the level of ureter obstruction and ureter entrapment in the inguinal hernia.^[1]^ The majority of the cases in the literatures describe the insertion of a percutaneous nephrostomy tube before surgery to prevent any more graft loss.^[1–4,6–16]^ Antegrade pyelography can further confirm the presence of hydroureteronephrosis and ureter entrapment in the hernial sac; however, the clinical management does not change.

In the present case, the DJ was inserted in the antegrade direction preoperatively, which significantly enabled identification of the ureter during surgical exploration of the inguinal canal. In our case, the ureter was located deep in the posterior wall of the inguinal canal and close to the neck of the hernial sac. To our knowledge, this is one of the few studies to have preoperative DJ insertion.^[17]^ Although most of the case reports related to this topic had no DJ insertion prior to surgery, there is no data regarding their operation time and the risk of ureter injury during surgical exploration. We propose that preoperative ureteral stent insertion may be a good option to avoid ureteral injury during herniorrhaphy of the transplant ureter inguinal hernia.

## 4 Conclusion

Transplant ureter inguinal hernia is a rare condition that leads to graft hydronephrosis and subsequent acute renal failure. General surgeons and urologists should consider ureteral inguinal hernia in the differential diagnosis of cases with a previous history of renal transplantation. Furthermore, transplant surgeons should understand the importance of orientation of the graft kidney. The orientation not only affects the graft vessel anastomosis but also the susceptibility of the ureter to compression by the hernial sac.

## Acknowledgment

The authors thank the patient and his family for permitting use of the medical data and other information that led to successful completion of the present article.

## Author contributions

**Resources:** Ping-Chin Lai.

**Supervision:** Wei-Ching Lin, Chao-Hsiang Chang.

**Writing – original draft:** Tian You Chang.

## References

[1] Cheung F Debartolo MM Copertino LM. Different management options for transplant ureteral obstructions within an inguinal hernia. Case Rep Transplant 2016;2016:4730494. 27144049 10.1155/2016/4730494 PMC4842045

[2] Hakeem AR Gopalakrishnan P Dooldeniya MD Irving HC Ahmad N. Inguinal herniation of a transplant ureter: lessons learned from a case of “water over the bridge”. Exp Clin Transplant 2016;14:103–5. 26114341 10.6002/ect.2014.0082

[3] Ciancio G Burke GW Nery J Huson H Coker D Miller J. Positional obstructive uropathy secondary to ureteroneocystostomy herniation in a renal transplant recipient. J Urol 1995;154:1471–2. 7658562

[4] Ingber MS Girdler BJ Moy JF Frikker MJ Hollander JB. Inguinal herniation of a transplant ureter: rare cause of obstructive uropathy. Urology 2007;70:01–3. 10.1016/j.urology.2007.09.054 18158065

[5] Pourafkari M Ghofrani M Riahi M. Inguinal herniation of a transplant kidney ureter: a case report. Iran J Radiol 2013;10:48–50. 10.5812/iranjradiol.10251 PMC3618907 23599715

[6] Bosmans I De Boe V Wissing KM Vanhoeij M Jacobs-Tulleneers-Thevissen D. A preventable cause of transplant hydroureteronephrosis: inguinal herniation of the transplant ureter: case report and review of the literature. Acta Chir Belg 2019;01–6. 10.1080/00015458.2019.1689650 31690216

[7] Osman Y Ali-El-Dein B El-Leithy R Shokeir A. Sliding hernia containing the ureter--a rare cause of graft hydroureteronephrosis: a case report. Transplant Proc 2004;36:1402–4. 15251343 10.1016/j.transproceed.2004.05.008

[8] Weingarten KE D’Agostino HB Dunn J Steiner RW. Obturator herniation of the ureter in a renal transplant recipient causing hydronephrosis: perioperative percutaneous management. J Vasc Interv Radiol 1996;7:939–41. 8951764 10.1016/s1051-0443(96)70874-6

[9] Sánchez AS Tebar JC Martín MS. Obstructive uropathy secondary to ureteral herniation in a pediatric en bloc renal graft. Am J Transplant 2005;5:2074–7. 15996264 10.1111/j.1600-6143.2005.00973.x

[10] Azhar R Boutros M Hassanain M. A rare case of obstructive uropathy in renal transplantation: ipsilateral indirect inguinal herniation of a transplant ureter. Transplantation 2009;88:1038–9. 19855251 10.1097/TP.0b013e3181b9e28d

[11] Odisho AY Freise CE Tomlanovich SJ Vagefi PA. Inguinal herniation of a transplant ureter. Kidney Int 2010;78:115. 20551935 10.1038/ki.2010.65

[12] Tran D Gaboriault J Collette S. Obstructive uropathy caused by an inguinal hernia in a kidney transplant recipient: Report of hernia cure by the shouldice technique. Dialysis Transplantation 2011;40:413–4.

[13] Mukha RP Devasia A Thomas EM. Ureteral herniation with intermittent obstructive uropathy in a renal allograft recipient. Urol J 2011;8:98. 21656483

[14] Youssef F Brown P Tappenden J Hall J Salim F Shrestha B. Obstructive uropathy secondary to incisional herniation of a transplant ureter - case report and review of literature. Ann Transplant 2013;18:53–6. 23792501 10.12659/AOT.883813

[15] Esposito M Ratnasekera A Sebastian E Youssef N. Femoral herniation of transplanted ureter after deceased-donor kidney transplantation. Int J Surg Case Rep 2015;10:115–7. 25828475 10.1016/j.ijscr.2015.03.026 PMC4430182

[16] Anderson E Corcoran A. Obstructive uropathy due to an incarcerated ureteroinguinal hernia. World J Nephrol Urol 2015;4:237–9.

[17] Bugeja A Clark EG Sood MM Ali SN. As in real estate, location is what matters: a case report of transplant ureteral obstruction due to an inguinal hernia. Can J Kidney Health Dis 2018;5:01–4. 10.1177/2054358117753620 PMC5774734 29372065

